# 552. Biodegradable Temporizing Matrix Soaked in Epinephrine Saline Solution for Improved Hemostasis

**DOI:** 10.1093/jbcr/irag033.249

**Published:** 2026-04-06

**Authors:** Paige Wells, Alyssa D Brown, Alan Pang

**Affiliations:** Texas Tech University Health Sciences Center, Lubbock, TX; Texas Tech University Health Sciences Center, Lubbock, TX; Texas Tech University Health Sciences Center, Lubbock, TX

## Abstract

**Introduction:**

Biodegradable temporizing matrix is a synthetic product that facilitates growth of neodermis over burned tissue providing a suitable wound bed for future skin grafting. Second and third degree burns to the back, in major burns, are a large area of tissue coverage, and it is often a more difficult to graft area due to the size and decreased air flow to the area. The back often bleeds during debridement leading to the necessity of multiple transfusions for the patient. We hypothesize that biodegradable temporizing matrix soaked in epinephrine saline solution decreases the amount of transfused blood products.

**Methods:**

This study was approved by the Texas Tech IRB Committee. We analyzed results from 3 patients who had back grafting with biodegradable temporizing matrix soaked briefly in epinephrine saline solution to saturation. The epinephrine saline solution was 1:100000 epinephrine to saline solution. The biodegradable temporizing matrix was then applied to the burned tissue after debridement. No additional adhesive was applied. The primary outcome measures were qualitative biodegradable temporizing matrix adherence and volume of blood products transfused compared to patients who received biodegradable temporizing matrix alone. Secondary outcome measures included post-operative and pre-operative hemoglobin and platelet comparison. All statistical analysis was performed by GraphPad Prism (v10.0.0).

**Results:**

Patients who received the epinephrine saline solution soaked biodegradable temporizing matrix required 2 fold less blood products intra-operatively and post-operatively compared to non-epinephrine soaked biodegradable temporizing matrix grafting. The patients with epinephrine saline solution soaked biodegradable temporizing matrix had significantly more graft adherence and decreased intraoperative time.

**Conclusions:**

Pre-soaking biodegradable temporizing matrix in epinephrine saline solution significantly reduced the total amount of blood product transfusion post-operatively. We postulate that the reduction in overall amount of blood products required was due to limiting blood loss by improving biodegradable temporizing matrix adhesion.

**Applicability of Research to Practice:**

Pre-soaking biodegradable temporizing matrix in epinephrine saline solution may help decrease the amount of blood products transfused in the perioperative and post-operative period, reducing resource utilization without compromising patient outcomes.

**Funding for the study:**

N/A.

